# Anaerobic methane‐oxidizing activity in a deep underground borehole dominantly colonized by *Ca* . Methanoperedenaceae

**DOI:** 10.1111/1758-2229.13146

**Published:** 2023-02-13

**Authors:** Hiroki Nishimura, Mariko Kouduka, Akari Fukuda, Toyoho Ishimura, Yuki Amano, Hikari Beppu, Kazuya Miyakawa, Yohey Suzuki

**Affiliations:** ^1^ Department of Earth and Planetary Science The University of Tokyo Tokyo Japan; ^2^ Graduate School of Human and Environmental Studies Kyoto University Kyoto Japan; ^3^ Horonobe Underground Research Center Japan Atomic Energy Agency Horonobe‐cho, Hokkaido Japan; ^4^ Nuclear Fuel Cycle Engineering Laboratories Japan Atomic Energy Agency Ibaraki Japan

## Abstract

The family *Ca*. Methanoperedenaceae archaea mediates the anaerobic oxidation of methane (AOM) in different terrestrial environments. Using a newly developed high‐pressure laboratory incubation system, we investigated 214‐ and 249‐m deep groundwater samples at Horonobe Underground Research Laboratory, Japan, where the high and low abundances of *Ca*. Methanoperedenaceae archaea have been shown by genome‐resolved metagenomics, respectively. The groundwater samples amended with ^13^C‐labelled methane and amorphous Fe(III) were incubated at a pressure of 1.6 MPa. After 3–7 days of incubation, the AOM rate was 45.8 ± 19.8 nM/day in 214‐m groundwater. However, almost no activity was detected from 249‐m groundwater. Based on the results from 16S rRNA gene analysis, the abundance of *Ca*. Methanoperedenaceae archaea was high in the 214‐m deep groundwater sample, whereas *Ca*. Methanoperedenaceae archaea was undetected in the 249‐m deep groundwater sample. These results support the in situ AOM activity of *Ca*. Methanoperedenaceae archaea in the 214‐m deep subsurface borehole interval. Although the presence of Fe‐bearing phyllosilicates was demonstrated in the 214‐m deep groundwater, it needs to be determined whether *Ca*. Methanoperedenaceae archaea use the Fe‐bearing phyllosilicates as in situ electron acceptors by high‐pressure incubation amended with the Fe‐bearing phyllosilicates.

## INTRODUCTION

Anaerobic oxidation of methane (AOM), a metabolism performed by microorganisms, including anaerobic methanotrophic archaea (ANME) inhabiting deep seafloor sediments, has been long examined (Boetius et al., 2000; Hinrichs et al., 1999). AOM is a metabolic pathway theoretically predicted to be pressure dependent, given the increased dissolved methane concentration with pressure (Timmers et al., 2015). Experimentally, the positive correlation between incubation pressure and AOM rate from deep‐sea sediments enriched with ANME has been demonstrated by pressure‐variable incubation systems (Krüger et al., 2008; Nauhaus et al., 2002). In the terrestrial subsurface, metabarcoding and metagenomic analyses have shown the dominance of the family *Candidatus* (*Ca*.) Methanoperedenaceae (formerly ANME‐2d; e.g., Flynn et al., 2013; Miettinen et al., 2018). Some members of *Ca*. Methanoperedenaceae are dominantly found in near‐surface environments using versatile electron acceptors, such as nitrate (Haroon et al., 2013), Fe(III) (Cai et al., 2018), and Mn(IV) (Leu, Cai, et al., 2020). In contrast to the near‐surface members demonstrated for their AOM activities by incubation experiments under ambient pressure, subsurface members of *Ca*. Methanoperedenaceae have been poorly characterized for their AOM activities.

The Horonobe Underground Research Laboratory (URL) was constructed in northern Hokkaido, Japan. Our previous metagenomic study of groundwater from a 214‐m deep underground borehole has shown abundant *Ca*. Methanoperedenaceae archaea (Hernsdorf et al., 2017). The near‐complete genome of *Ca*. Methanoperedenaceae reconstructed from the 214‐m deep groundwater was equipped with a set of genes needed for AOM and potentially involved in the reduction of Fe(III) and Mn(IV) (Ettwig et al., 2016; Kletzin et al., 2015). In contrast, the abundant *Ca*. Methanoperedenaceae was nearly undetected in a 249‐m deep underground borehole of the URL, where the groundwater has similar hydrogeochemical features with those of the 214‐m deep groundwater. Thus, the factors controlling the abundance and activity of *Ca*. Methanoperedenaceae archaea are still largely unknown.

In this study, first, AOM rates were measured under high‐pressure conditions by extending previously established incubation experiments with ^13^C‐labelled methane (Ino et al., 2018). Then, 16S rRNA gene amplicon analysis was conducted using the 214‐m and 249‐m deep groundwater samples to verify the clear difference in the abundance of the *Ca*. Methanoperedenaceae archaea reported in our previous study (Hernsdorf et al., 2017). As a result, the AOM rates were higher in the 214‐m deep groundwater sample than in the 249‐m deep groundwater sample, which correlates with the abundance of *Ca*. Methanoperedenaceae archaea, shown by 16S rRNA gene amplicon analysis.

## RESULTS

### Study site and sampling

The Horonobe area is located on the eastern margin of a Neogene to Quaternary sedimentary formations. In September 2021, groundwater samples were obtained from two boreholes drilled in the Koetoi Formation (Neogene to Quaternary diatomaceous mudstones). A borehole named 08‐E140‐C01 was drilled diagonally downward with a vertical angle of −45° from the 140‐m gallery at Horonobe URL (45°02′43″ N, 141°51′34″ E). The groundwater sample obtained from an interval at an average depth of 214‐m below ground level (m b.g.l.) within the 08‐E140‐C01 borehole is referred to as ESB214. The other borehole, 09‐V250‐M02, was drilled horizontally from the 250‐m gallery. The groundwater sample obtained from an interval at a depth of 249‐m b.g.l. is referred to as VSB249. The temperature and hydrostatic pressure are 18–20°C (Table S1) and around 1.6 MPa (Mezawa et al., 2017). The geochemical features of groundwater are characterized by neutral pH, salinity lower than seawater, and relatively high concentrations of total organic carbon around 20–50 mg/L (Miyakawa et al., 2020). The groundwater samples contain methane as a main dissolved gas component (90%–100%; Miyakawa et al., 2017).

Multi‐packer systems were used to collect groundwater from the target intervals (Nanjo et al., 2012). A pressure‐resistant stainless filter holder with a membrane filter was directly connected to the tubing outlet to obtain microbial cells without lowering the hydrostatic pressure. In situ hydraulic pressure was retained inside the filter holder during the refrigerated shipment to the laboratory. In the supplementary material, the detailed sampling procedures are described.

### AOM activities in borehole groundwater samples

**FIGURE 1:**
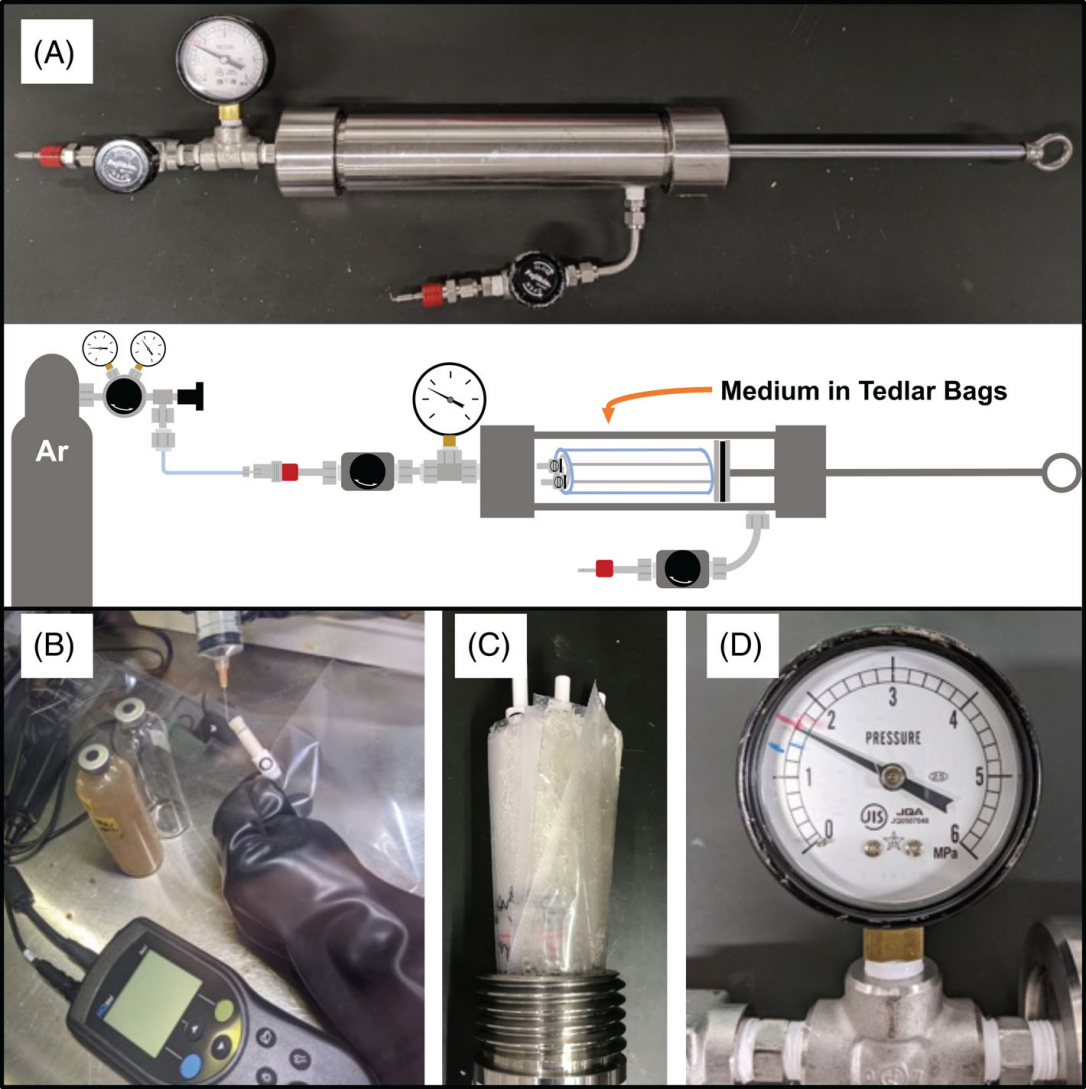
Graphical descriptions of high‐pressure incubation experiments. A picture and schematic illustration of the high‐pressure incubation system indicating the inner vessel structure and the overall experimental setup (A). A picture showing medium preparation using a Tedlar bag inside a glovebox (B). The insertion of Tedlar bags into a stainless vessel (C) and a pressure gage measuring an internal vessel pressure of 1.6 MPa (D).

For the rate measurements of AOM, a high‐pressure incubation system was newly developed to reproduce high‐pressure conditions. The system comprised a stainless vessel, a piston, a pressure gauge, valves, and gas lines (Figure 1A). The microbial cells originally suspended in groundwater were collected using filtration, some portions of which were subjected to 16S rRNA gene amplicon analysis. The cells were amended with either electron acceptors, namely, amorphous Fe(III), nitrate or sulfate. The prepared cultures were incubated in Tedlar bags to avoid direct contact with the vessel material (Figure 1B,C). The inside pressure during incubation was set as a hydraulic pressure of 1.6 MPa (Figure 1D). The alterations in carbon stable isotopic ratio (*δ* ^13^C) in DIC and alkalinity during incubation were measured to calculate the AOM rates. By dividing microbial cell densities in cultures at the beginning of the incubation by those in in the original Horonobe groundwater samples, the concentration factors were determined to be 7.2 and 3.2 times for ESB214 and VSB249, respectively. The concentration factors for ESB214 and VSB249 were not controlled to be the same, because the microbial cell densities were measured after the incubation experiments.

**FIGURE 2:**
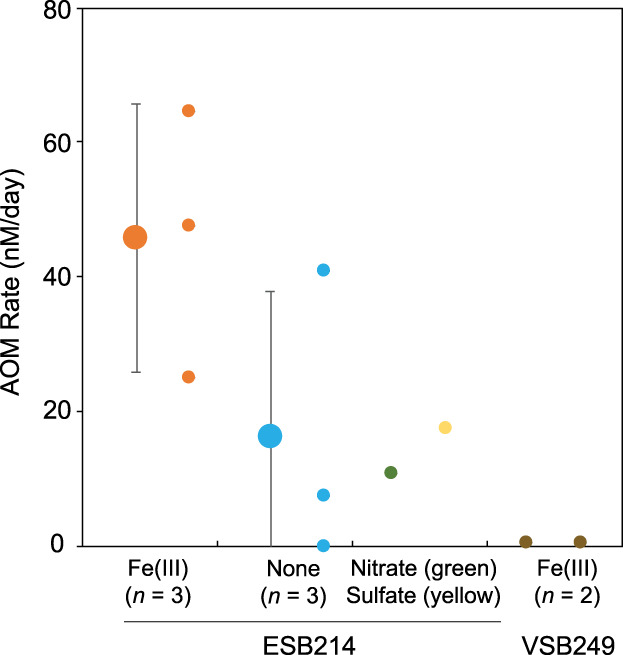
Anaerobic oxidation of methane (AOM) rates collected from high‐pressure incubation experiments. Smaller‐filled circles indicate AOM rates for single experiments, and larger ones with error bars show the means and standard deviations of the replicates. The amended electron acceptors and replication numbers (*n*) are labelled with the circles and groundwater sample IDs.

As a result, amorphous Fe(III) amendment using ESB214 showed relatively high AOM rates at 45.8 ± 19.8 nM/day (Figure 2). ESB214 was incubated either with nitrate, sulfate or without any electron acceptor. These incubation experiments showed the comparative level of AOM rates with Fe(III) amendment experiments (Figure 2). In terms of VSB249, amorphous Fe(III) amendment was associated with nearly undetected levels of AOM activities.

### The 16S rRNA gene sequence analysis of microbial communities before and after the incubation

**FIGURE 3:**
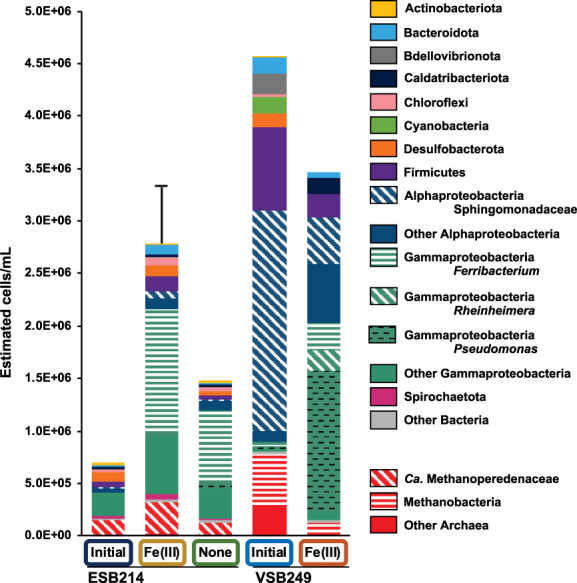
Microbial compositions of ESB214 and VSB249 before and after incubation with and without Fe(III) amendment based on 16S rRNA gene sequence analysis. Each colour represents major taxonomic groups ranging from the genus to phylum level. Abundant taxonomic groups were highlighted with shading. The vertical axis corresponds to the average cell density measured by direct cell counting of single, duplicate, or triplicate samples. Each band shows the proportions of taxonomic groups normalized by the average cell density. The error bar shows the standard deviation of triplicated cell counting.

**FIGURE 4:**
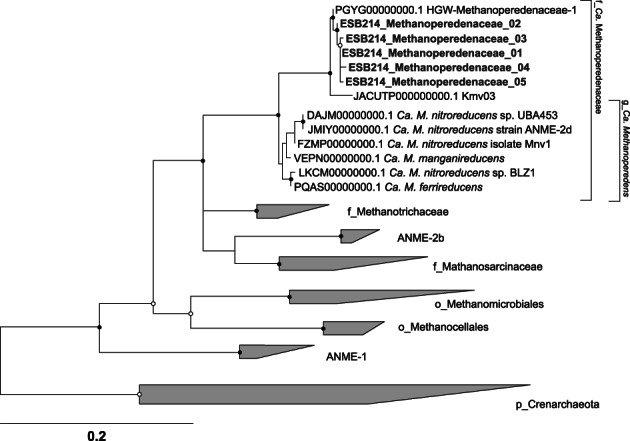
Neighbour‐joining phylogenetic tree including the *Ca*. Methanoperedenaceae‐affiliated sequences obtained before and after incubation experiments and closely related sequences from public databases. Regarding sequences from this study, only the top five most abundant sequences before and after incubation experiments were shown and written in blue. This tree was rooted in *Escherichia coli* (not shown). The 1000 bootstrap replicates were collected with a maximum likelihood method, and values >75% and >90% are indicated by open and filled circles at branches, respectively. The scale bar shows 0.2 expected changes per site.

Before and after the short‐term incubation, microbial communities were assessed using 16S rRNA gene sequence analysis (Figure 3). Microbial compositions after ESB214 incubation with nitrate and sulfate were not obtained, due to the lack of PCR amplification. The family *Ca*. Methanoperedenaceae was the most abundant at the family level in ESB214 at the beginning of the incubation, accounting for 17% of the total high‐quality reads (labelled as ‘ESB214_initial’ in Figure 3). In contrast, the family *Ca*. Methanoperedenaceae was not detected in VSB249 before and after the incubation (labelled as ‘VSB249’ in Figure 3). This result is consistent with the dominance and absence of *Ca*. Methanoperedenaceae in ESB214 and VSB249, previously reported by Hernsdorf et al. (2017). After ESB214 incubation with and without Fe(III) amendment, the abundance of *Ca*. Methanoperedenaceae was nearly maintained (Figure 3). The phylogenetic relationships of *Ca*. Methanoperedenaceae‐affiliated sequences were characterized in a neighbour‐joining tree (Figure 4). The obtained sequences are closely related to metagenome‐assembled genomes (MAGs): HGW‐Methanoperedenaceae‐1 reconstructed from ESB214 (Hernsdorf et al., 2017) and Kmv03 reconstructed from an active mud volcano (Mardanov et al., 2020). None of the obtained sequences from ESB214 and VSB249 were affiliated with a bacterial anaerobic methanotroph, *Ca*. *Methylomirabilis oxyfera* (Ettwig et al., 2010).

Most of the microbial community in VSB249 was affiliated with Alphaproteobacteria, in which the genus *Sphingomonas* accounted for 54% of the total high‐quality reads (Figure 3). *Sphingomonas* spp. known to degrade recalcitrant organic matter have been widely reported from the deep subsurface (Fredrickson et al., 1995, 1999). Instead of *Ca*. Methanoperedenaceae archaea in ESB214, the members of Methanobacteriaceae were the dominant archaeal populations in VSB249.

After the incubation experiments, bacterial compositions changed dramatically. During ESB214 incubation experiments with and without Fe(III) amendment, the genus *Ferribacterium* of Gammaproteobacteria increased to ~40% of the total high‐quality reads (Figure 3). The genus *Ferribacterium* is known as Fe(III) reducers and obligate anaerobes (Cummings et al., 1999). In terms of VSB249, the gammaproteobacterial proportion mainly comprised the genera *Ferribacterium*, *Rheinheimera*, and *Pseudomonas* increased (Figure 3). The genus *Rheinheimera* has been reported from the deep terrestrial subsurface, including VSB249 (Ise et al., 2017). Since the genus *Rheinheimera* is aerobic (Brettar et al., 2002), its presence in VSB249 might be from drilling disturbance introducing O_2_. A high proportion of *Pseudomonas* spp. has been reported from 281 to 312‐m deep groundwater from a borehole drilling from the surface near the underground facility (Kato et al., 2009). As some members of the genus *Pseudomonas* mediate heterotrophic Fe(III) reduction, amorphous Fe(III) might be favourable for *Pseudomonas* growth (Arnold et al., 1988; Naganuma et al., 2006).

### Characterization of suspended particulates in groundwater samples

**FIGURE 5:**
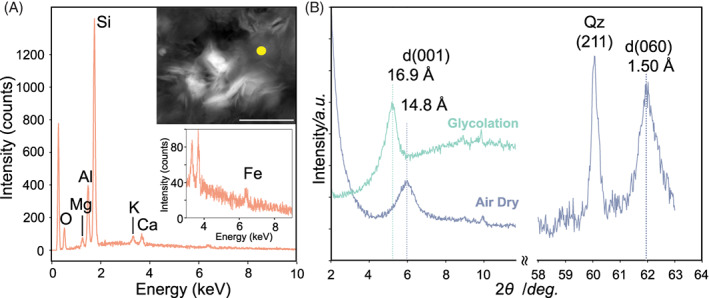
Mineralogical features of suspended particulates in ESB214. An energy dispersive x‐ray spectrum of the particulates was obtained from a yellow circle in a back‐scattered electron image (A). The scale bar in an electron microscopy image indicates 3.0 μm. An enlarged spectrum emphasizing a Fe peak is also shown. Powder XRD pattern of the particulates (B). Low‐angle XRD patterns (2*θ*: 2°–12°) of air‐dried and ethylene glycol‐sprayed samples for clarifying the expansion of basal spacings. High‐angle pattern (2*θ*: 58°–63°) for the air‐dried sample, including (060) reflection of phyllosilicate minerals for distinguishing dioctahedral and trioctahedral sheet structures.

The AOM rate measurements without amendments of the electron acceptors, such as nitrate, sulfate, and amorphous Fe(III) showed the AOM mediation in ESB214. Given the high concentration of total iron relative to that of Fe(II), Fe(III) might be present in ESB214 (Table S1). As Fe(III) is likely present as insoluble Fe(III) species under reducing subsurface conditions rather than soluble Fe(III) species, Fe(III)‐bearing particles that are not eliminated by filtration before the groundwater analysis might be present in ESB214. Additionally, the loading of some yellowish‐brown material on the filters was visually recognized after the passage of ESB214 (6.8 L). In contrast, the filters after the passage of VSB249 (64 L) gave no apparent loading on the filters. There appears to be the potential that this yellowish‐brown material is an essential factor controlling the activity of AOM by *Ca*. Methanoperedenaceae. Thus, mineralogical characterization of the yellowish‐brown material was performed. Scanning electron microscopy equipped with energy dispersive spectrometry (SEM‐EDS) and x‐ray diffractometry (XRD) analyses were conducted to identify mineral phases in the yellowish‐brown material. The combination of XRD and SEM‐EDS analyses is necessary to identify a mineral phase, because XRD analysis provides no information on the chemical composition but the crystal structure. Instead, SEM‐EDS analysis provides no information on the crystal structure but the chemical composition of the material. The obtained EDS spectrum showed the elemental composition corresponding to a smectite mineral in association with a trace amount of iron (Figure 5A). Consistently, the obtained XRD pattern also indicated the peak of smectite basal spacing and its shift after glycolation treatment showing the expansion of *d*_001_ were verified in XRD patterns (Figure 5B). The peak of smectite (060) reflection in the higher angle pattern suggests a dioctahedral sheet structure, which tends to retain trivalent cations, such as Fe(III) (Brigatti et al., 2006).

## DISCUSSION

### *AOM activities potentially mediated by* Ca*. Methanoperedenaceae*

Genomic profiling of *Ca*. Methanoperedenaceae indicates a complete gene set for reverse methanogenesis and related cofactor enzymes (Leu, McIlroy, et al., 2020). Additionally, various electron acceptors are reported for *Ca*. Methanoperedenaceae, including nitrate (Haroon et al., 2013), nitrite (Arshad et al., 2015), Fe(III) (Cai et al., 2018; Ettwig et al., 2016), Mn(IV) (Ettwig et al., 2016; Leu, Cai, et al., 2020), and Cr(VI) (Lu et al., 2016). HGW‐Methanoperedenaceae‐1 and Kmv03 are not equipped with genes involved in nitrate reduction but heme‐rich multiheme *c*‐type cytochromes (MHCs) for extracellular electron transfer. Remarkably, HGW‐Methanoperedenaceae‐1 and Kmv03 are equipped with 11 and 18 copies of MHCs, in which the maximum numbers of heme‐binding motifs (CXXCH) in a single MHC gene are >50 and 22, respectively. Many MHCs with high numbers of heme‐binding motifs are commonly found in bacterial groups, including the family Shewanellaceae and Geobacteraceae, for the electron transfer to solid metal oxides for their growth (Methé et al., 2003; Shi et al., 2007, 2016). From these genomic features, the *Ca*. Methanoperedenaceae members, represented by 16S rRNA gene sequences from this study, are estimated to be capable of electron transfer to iron‐bearing insoluble electron acceptors.

In this study, there was an attempt to measure AOM rates under high‐pressure conditions using a newly developed laboratory incubation system. The incubation was set to mimic in situ conditions as much as possible. A factor slightly different from the in situ counterparts was the cell densities, which were 7.2 and 3.2 times higher in the incubation media than those originally in ESB214 and VSB249, respectively. Although the higher cell density might enhance necromass degradation, this effect was reduced by keeping the incubation time short. Nevertheless, there is the potential that the AOM rates were underestimated, given that other Fe(III)‐reducing activities (e.g. *Ferribacterium* spp.) are competitive with electron acceptors available in the incubation media. Additionally, some of the incubation conditions differ from in situ, namely, the absence of rock matrix, the lower concentration of nutrients originally contained in groundwater. Therefore, the obtained rates should be interpreted as the potential rates of the subsurface AOM.

Our incubation experiments obtained the highest AOM rates when amorphous Fe(III) was added to the incubation media prepared from ESB214. The Fe(III)‐dependent AOM of *Ca*. Methanoperedenaceae archaea in ESB214 was hypothesized in our previous study, given that canonical correspondence analysis statistically clarified the relationship between the high concentration of total iron and the high abundance of *Ca*. Methanoperedenaceae (Hernsdorf et al., 2017). This relationship is also supported by results from *Ca*. Methanoperedenaceae‐dominated bioreactor experiments, which showed ferrihydrite‐dependent AOM activities (Cai et al., 2018; Ettwig et al., 2016). Therefore, the AOM activities observed in the incubation experiments serve as the first direct evidence that *Ca*. Methanoperedenaceae mediates Fe‐dependent AOM under high‐pressure conditions. This notion is also supported by the dominance of *Ca*. Methanoperedenaceae in the original ESB214 as a sole anaerobic methanotroph in the microbial community.

### Comparison of the AOM rates between near‐surface and subsurface habitats

In this study, we successfully quantified AOM rates mediated by the groundwater community, including the deep subsurface *Ca*. Methanoperedenaceae members. The average AOM rate of the Fe‐amended incubation experiment using ESB214 (45.8 ± 19.8 nM/day) is equivalent to the cell‐specific AOM rate of 0.25 ± 0.13 pM/day, which was calculated by dividing the rates with the *Ca*. Methanoperedenaceae cell number. In no‐amended or sulfate/nitrate amended systems, the AOM rates were much lower than that in the Fe‐amended system.

The reported cell‐specific AOM rates from *Ca*. Methanoperedenaceae range 10–10^2^ μM/day from near‐surface environment lineages (Cai et al., 2018; Guerrero‐Cruz et al., 2018; Haroon et al., 2013; Leu, Cai, et al., 2020; Vaksmaa et al., 2017). The cell‐specific rate in ESB214 (0.25 ± 0.13 pM/day) is much slower than that mediated by the surface lineages. Our previous study obtained an cell‐specific AOM rate of 0.01 pM/day from granite‐hosted deep subsurface lineage (Ino et al., 2018). Therefore, the obtained AOM rates are reasonable for ESB214. The slower rate in the deep granite aquifer might be explained by the low level of methane (several hundred μM), in contrast to the abundant methane in Horonobe sedimentary rocks (several mM; Table S1).

### Potential in situ electron acceptors for AOM

AOM activities measured by the incubation experiments are considered to reflect AOM activities in the boreholes. Nearly undetected AOM activity from the incubation experiments using VSB249 could be from the absence of Ca. Methanoperedenaceae members shown from the 16S rRNA gene amplicon analysis. In contrast, moderately high AOM activities measured by the incubation experiments might reflect the abundance of *Ca*. Methanoperedenaceae members who were maintained through the incubation experiments using ESB214. To explain the different levels of AOM activities, it is crucial to clarify electron acceptors such as Fe(III) available in the borehole containing ESB214 to understand the factors controlling subsurface AOM activities.

Given the low stability of amorphous Fe(III) in the reducing conditions of ESB214, it is likely that the particulate components other than amorphous Fe(III) could be used as insoluble electron acceptors for AOM activities. Indeed, the presence of dioctahedral smectite associated with iron was verified using XRD and EDS analyses of ESB214 suspended particulates (Figure 5). Thus, detected levels of AOM activities without any amendment of electron acceptors were explained in ESB214 (‘None’ in Figure 3). Similarly, AOM rates were also reasonable with the amendment of nitrate or sulfate (Figure 3), given the availability of the dioctahedral smectite in ESB214. Although the use of nitrate and sulfate has been addressed for the respiration of the *Ca*. Methanoperedenaceae members (Haroon et al., 2013; Ino et al., 2018; Weber et al., 2017), these electron acceptors are scarce in Horonobe groundwater samples (Table S1). Hence, it is likely that equivalent AOM rates obtained from nitrate‐ or sulfate‐amended incubation experiments were also dependent on particulates originally present in the borehole.

The importance of the particulates was indirectly evaluated for VSB249. The particulates were not visible on a 47‐mm diameter filter after filtering a large volume of the VSB249 groundwater. In this study, we did not detect any *Ca*. Methanoperedenaceae members from VSB249 might result from the lack of particulates. As expected, the AOM rate measurements of VSB249 showed the slowest AOM rates even with the amendment of amorphous Fe(III). This result highlights the importance of the particulates for the subsurface AOM activities.

From mineralogical analyses of the particulates, Fe‐associated smectite was detected. Thus, it is proposed that the smectite is a candidate electron acceptor used for AOM in the groundwater. Still, it is unknown whether *Ca*. Methanoperedenaceae archaea can use Fe(III) in phyllosilicate minerals. Previously, the capability of Fe(III) reduction in smectite minerals has been shown for the bacterial groups with high numbers of heme‐rich MHC genes in their genomes, such as the genera *Shewanella* and *Geobacter* (Dong et al., 2009, and references therein). Thus, the possibility of electron transfer to Fe(III) in the smectite clay present in the particulates by the members of *Ca*. Methanoperedenaceae equipped with a higher number of heme‐rich MHC genes, will be investigated.

### Implications for the geological disposal of nuclear waste

AOM activities coupled with Fe(III) reduction result in the abiotic reduction of U(VI) by biologically reduced iron (Wu et al., 2010). Likewise, ^99^Tc, a long‐lived fission product of ^235^U, is reduced by Fe(II) (De Luca et al., 2001). Thus, the subsurface migration of long‐lived radionuclides might be influenced by AOM activity showed in the borehole. The stimulation of AOM activities coupled with Fe(III) reduction after back‐filling deep repositories for high‐level nuclear wastes causes the establishment of reducing conditions and the retardation of radionuclide migration.

## CONCLUSION

In this study, a combination of high‐pressure incubation using ^13^C‐labelled methane and 16S rRNA gene amplicon analysis clarified AOM activity in *Ca*. Methanoperedenaceae‐abundant groundwater amended with amorphous Fe(III). Without the amendments of electron acceptors, AOM activity was low but experimentally detectable. The AOM activity without the amended electron acceptors might be explained by the presence of Fe‐bearing phyllosilicates as suspended particles in the groundwater. Our future high‐pressure incubation experiments will be performed by using the Fe‐bearing phyllosilicates to determine in situ electron acceptors for subsurface AOM activity.

## AUTHOR CONTRIBUTIONS

Y.S. and H.N. designed the study. K.M., Y.S. and H.N. collected groundwater samples. H.N. and A.F. performed high‐pressure incubation experiments. T.I. and H.N. measured stable carbon isotopes. H.N. performed XRD and SEM‐EDS analyses. M.K., H.B., Y.A. and H.N. conducted 16S rRNA gene sequence analysis. H.N. and Y.S. co‐wrote the manuscript. All authors discussed the results and commented on the manuscript.

## CONFLICT OF INTEREST

The authors declare that there is no conflict of interest.

## Supporting information


**Appendix S1:** Supplementary Information

## Data Availability

The data regarding this study are available from the corresponding author upon request.
