# Supplementary material for: Anaerobic methane‐oxidizing activity in a deep underground borehole dominantly colonized by Ca . Methanoperedenaceae
Source: Environ Microbiol Rep. 2023 Feb 13;15(3):197–205. doi: 10.1111/1758-2229.13146 (PMC10464669; doi:10.1111/1758-2229.13146)
Supplement: Supplementary file 1 — Appendix S1: Supplementary Information [file EMI4-15-197-s001.docx]

Supporting Information

**Materials and Methods**

*Site description and biological sampling*

The Horonobe URL was constructed by Japan Atomic Energy Agency with the objectives of research and development of geological disposal technologies of high-level radioactive waste. The sediments around the URL consist of the Wakkanai Formation (Neogene siliceous mudstones) overlain by the Koetoi Formation (Neogene to Quaternary diatomaceous mudstones). The origin of ESB214 and VSB249 is fossil seawater mostly diluted by meteoric water (Teramoto *et al.*, 2006; Iwatsuki *et al.*, 2009). The geochemical characteristics were investigated in previous studies and summarized in Table S1. Groundwater samples were filtered with a membrane filter (type GPWP, 0.22-µm pore size: Merck Millipore, Darmstadt, Germany) in a pressure-resistant stainless filter holder (HP Filter Holder, 47 mm, stainless steel: Merck Millipore). The amount of filtered groundwater volume were 17.3 L and 64 L for ESB214 and VSB249, respectively. Filtered groundwater samples were obtained from the filter holder directly into Ar-flushed 100-ml glass vials. The filter holders and the filtered groundwater were stored at 4 ℃. All groundwater and microbial samples collected between September 8th and 10th, 2021 were used for incubation experiments within one week after sampling.

**Table S1.** Borehole and groundwater characteristics of Horonobe URL. All data except for Eh and dissolved gas are reported in Miyakawa *et al.* (2020).

|  | Depth interval | Temp. (℃) | pH | Eh  (mV) | Dissolved Gas |  | Cation, dissolved iron and anions (mg/L) | | | | | |
| --- | --- | --- | --- | --- | --- | --- | --- | --- | --- | --- | --- | --- |
|  | (m b.g.l.^a^) |  |  |  | CH_4_ (mmol/kg) |  | Na^+^ | Cl^-^ | Fe^2+^ | T.Fe^d^ | NO_3_^-^ | SO_4_^2-^ |
| ESB214 | 209.7 - 218.7 | 19.1 ± 1.0 | 7.6 ± 0.2 | -219^b^ | 19.5^c^ |  | 1633 ± 5 | 1418 ± 5 | 0.26 ± 0.26 | 0.60 ± 0.09 | 0.1 - 0.2 | < 0.1 |
| VSB249 | 248.8 - 248.9 | 19.5 ± 0.6 | 7.3 ± 0.1 | -226 ± 52^b^ | 2.7 ± 1.7^c^ |  | 1917 ± 15 | 1920 | 0.30 ± 0.02 | 0.32 ± 0.04 | < 0.1 | < 0.1 |

^a^meters below ground level.

^b^Data reported in Mezawa *et al.* (2018)

^c^Data reported in Miyakawa *et al.* (2017)

^d^Total iron concentration

*High-pressure incubation*

Stainless holders, in which *in situ* hydraulic pressure was retained for one week after on-site sampling, were opened in a glovebox filled with Ar gas. Microbial cells trapped on the membrane filter inside the stainless holders were suspended in autoclaved, deoxygenated 50 mM NaCl solution (pH 7.4). This NaCl concentration was selected to mimic the salinity of groundwater (Table S1). The original groundwater was not used as a base medium, because the high concentration of dissolved inorganic carbon (DIC) lowers the sensitivity of quantification of ^13^CO_2_ production. Alternatively, cultures were prepared by the ten-fold dilution of filter-sterilized groundwater samples with the 50 mM NaCl solution. Then, the cultures were amended either with 0.1 mM amorphous Fe(III), 0.5 mM Na nitrate or 0.5 mM Na sulfate for an electron acceptor. The low levels of O_2_ in the cultures were assured by adding stock FeCl_2_ solution for a final concentration of 2.5 mg/L (scavenge trace amounts of O_2_; Williamson and Coates 2013) and then monitored by a fiber-optic oxygen meter (MICROX TX3-TRACE; PreSens, Regensburg, Germany). Finally, cultures were amended with the cell suspensions and ^13^C-labeled methane (9 mM) at a concentration nearly equivalent to the groundwater samples (Table S1). Then, the cultures prepared inside of Tedlar bags were transferred into the stainless vessel and pressurized with Ar gas at a hydraulic pressure of 1.6 MPa. After three or seven days, the cultures were collected and subjected to chemical and microbiological characterizations.

The amorphous Fe(III) was synthesized by titrating 80 mM FeCl_3_ solution by 10 N NaOH and thoroughly rinsed with deionized water before amendment. ^13^C-labeled methane (CLM-3590; Cambridge Isotope Laboratories, Andover, MA, USA) was stored inside Tedlar bags for one week before amendment to remove trace amount of H_2_ contained as impurities.

The cell suspensions were prepared by resuspending cells in Ar-purged and autoclaved 50-mM NaCl solution (pH 7.4). In detail, the high-pressure filter holders were shipped to the laboratory at 4 ℃ after sampling. The holders were opened inside of an anaerobic glove box. The filters with suspended particulates and cells were then transferred to a plastic tube and thoroughly shaken with the NaCl solution. The cell suspension was transferred and stored inside an Ar-purged 50 mL glass vial sealed with a butyl rubber stopper and an aluminum cap. The same preparation procedure was applied to both ESB214 and VSB249. The suspensions were utilized soon after preparation to minimize the effects of exposure to ambient pressure.

The cultures inoculated with cell suspension were originally prepared in 100 mL glass vials sealed with butyl rubber stoppers and aluminum caps. After preparation other than methane amendment, Ar-gas purging for each vial was conducted repeatedly until the oxygen level became lower than the detection limit of the fiber-optic oxygen meter. Then, cultures were transferred into Ar-purged Tedlar bags inside a glove box, and the treated ^13^C-labeled methane was amended for each bag with a gas-tight syringe. The bags were transferred into a stainless vessel. The original gas phase in the stainless vessel was replaced and pressurized with Ar gas.

Some portions of inoculated cultures were fixed with 3.7 % formalin solution at neutral pH for cell counting and filtered with 0.22-µm pore-sized membrane filters. Filtrates were stored at 4℃ in blood-collection tubes sealed with thick butyl rubber stoppers to prevent air exchange and served for *δ*^13^C analysis. The remaining cultures were stored in plastic tubes for other chemical analyses at 4℃. The inoculated cultures were also processed at the beginning of the incubation.

*Chemical analysis procedures*

DIC concentrations were estimated by measuring alkalinity in the filtrates by acid titration using 0.02-N hydrochloric acid. Based on the amount of 0.02-N hydrochloric acid added into each culture and pH measured by a pH meter (LAQUA F-73, Horiba, Japan), alkalinity was calculated by a Gran plot method.

The filtrated samples stored inside of blood-collection tubes were transferred into glass vials sealed with butyl rubber stoppers and aluminum caps containing sulfamic acid. After the reaction with the acid, DIC species, mainly bicarbonate, were extracted as carbon dioxide. The *δ*^13^C of carbon dioxide were analyzed with an IsoPrime100 isotope ratio mass spectrometer (Isoprime Ltd., Cheadle Hulme, UK) equipped with a customized continuous-flow gas preparation system (MICAL3c; Ishimura *et al.*, 2004; 2008) at Kyoto University, Japan.

*Microscopic observations*

We conducted direct cell counting for measurement of total cell numbers in each incubated culture and original groundwater samples. In detail, 0.1 mL of fixed cell suspension or groundwater sample was filtrated by 0.22-µm pore size black polycarbonate filter (type GTBP; Merck Millipore) and stained with 10 × SYBR Green I (SYBR^®^ Green I Nucleic Acid Stain; Lonza, Rockland, ME, USA) in 1×TAE buffer for 3 min. at room temperature. Microbial cells on the filter were directly counted using an epifluorescence microscope (BX51; Olympus, Tokyo, Japan) equipped with a digital camera (DP70; Olympus). Two filters per sample were prepared and cell numbers were obtained by observation of 50 fields of view on a single specimen and calculation of their average.

*DNA extraction, sequencing and phylogenetic analysis*

Some portion of inoculated cultures before and after incubation were filtered on a 0.22-μm pore size filter (type GPWP; Merck Millipore) and subjected to DNA extraction using MO BIO'S PowerMax Soil DNA Isolation Kit (Qiagen, Inc., Valencia, CA, USA). 16S rRNA gene sequences were amplified by polymerase chain reaction (PCR) using LA *Taq* polymerase (TaKaRa-Bio, Inc., Kusatsu, Japan) for Illumina MiSeq paired-end sequencing. The primers Uni530F and Uni907R (Nunoura *et al.*, 2012) containing Illumina TruSeq adapter sequences (Illumina Inc., San Diego, CA, USA) were used for PCR. A reaction mixture was prepared in which the concentration of each oligonucleotide primer was 0.1 μM and that of the DNA template was ca. 0.1 ng/μL. Thermal cycling was performed with 35 cycles of denaturation at 96 °C for 20 s, annealing at 56 °C for 45 s, and extension at 72 °C for 120 s. The first PCR amplicon was used for the second PCR step, which was run with TruSeq P5 and Index-containing P7 adapters, and PCR program identical to the first, except with 10 cycles. The final PCR amplicon with the expected size was confirmed by electrophoresis on TAE (40 mM Tris acetate, 1 mM EDTA, pH 8.3) agarose gels (1 %), which was purified using a MinElute Gel Extraction Kit (Qiagen). 16S rRNA gene sequencing was performed using a MiSeq platform with MiSeq Reagent Kit v2 (Illumina).

After the removal of chimeras within BaseSpace (Illumina), the paired-end sequence reads were demultiplexed, trimmed and quality filtered using Cutadapt (Martin 2011) and further processed using DADA2 (Callahan *et al.*, 2016) to obtain consensus (Amplicon Sequence Variants; ASVs) sequences as phylotypes in QIIME2 (ver. 2022.2; Bolyen *et al.*, 2019). For alignment and taxonomic affiliation of ASVs, SINA Aligner (v1.2.11; Pruesse *et al.*, 2012) through the SILVA web interface (Pruesse *et al.*, 2007) and SILVA SSU Ref NR database (ver. 138) was used (Quast *et al.*, 2013). The neighbor-joining tree based on 16S rRNA gene sequences were constructed in the ARB software (Ludwig *et al.*, 2004) for *Ca.* Methanoperedenaceae-affiliated ASVs and closely related sequences retrieved from GenBank (http://www.ncbi.nlm.nih.gov/genbank/). Distantly related sequences were retrieved from the SILVA SSU Ref NR database (ver. 138) for the tree construction. Bootstrap analysis of the maximum-likelihood tree was performed with 1000 replicates using the IQ-TREE (multicore version 1.6.12; Trifinopoulos *et al.*, 2016). The prokaryotic ASVs obtained from ESB214 and VSB249 in this study were registered in DNA Data Bank of Japan (DDBJ) under the accession numbers TAAD01000001 to TAAD01000998.

*Mineralogical analyses for suspended particulates*

The phyllosilicate mineral characterization was performed by XRD pattern analysis. The suspended clay-sized particulates from ESB214 were obtained and vacuum-dried. The clay-sized particulates were mounted on a non-reflecting sample holder. The diffraction pattern was obtained with monochromatized Cu-Kα X-ray at an operation voltage of 40 kV and an operation current of 30 mA using an X-ray diffractometer (RINT-2100; Rigaku, Japan). After ethylene glycol was sprayed, the diffraction pattern of the wet sample was also obtained in addition to the dried sample. The 2*θ* range and resolution were 5 – 65° and 0.02°, respectively.

The portion of the dried clay-sized particulates were embedded in LR White Resin (London Resin Co., Ltd., Aldermaston, England) and solidified in an oven at 50 ℃ for 48 h. The resin block was coated with carbon (SC-701C Quick Carbon Coater; Sanyu Denshi Co., Ltd., Tokyo, Japan), and the morphological observation of suspended particulates and their elemental composition were obtained by a field emission SEM (S-4500; Hitachi) equipped with an EDS detector (Ultradry EDS detector NS7; Thermo Fischer Scientific Inc., Waltham, MA, USA). The analysis was operated at accelerating voltage of 15 kV and emission current of 13 µA.

**References**

Bolyen, E., Rideout, J.R., Dillon, M.R., Bokulich, N.A., Abnet, C.C., Al-Ghalith, G.A. *et al.* (2019) Reproducible, interactive, scalable and extensible microbiome data science using QIIME 2. Nat Biotechnol 37: 852-857.

Callahan, B. J., McMurdie, P.J., Rosen, M.J., Han, A.W., Johnson, A.J.A., and Holmes, S.P. (2016) DADA2: High-resolution sample inference from Illumina amplicon data. Nat Methods 13: 581-583

Ishimura, T., Tsunogai, U., and Gamo, T. (2004) Stable carbon and oxygen isotopic determination of sub-microgram quantities of CaCO_3_ to analyze individual foraminiferal shells. Rapid Commun Mass Sp 18: 2883-2888.

Ishimura, T., Tsunogai, U., and Nakagawa, F. (2008) Grain-scale heterogeneities in the stable carbon and oxygen isotopic compositions of the international standard calcite materials (NBS 19, NBS 18, IAEA-CO-1, and IAEA-CO-8). Rapid Commun Mass Sp 22: 1925-1932.

Iwatsuki, T., Ishii, E., and Niizato, T. (2009) Scenario development of long-term evolution for deep hydrochemical conditions in Horonobe area, Hokkaido, Japan. J Geogr 118: 700-716.

Ludwig, W., Strunk, O., Westram, R., Richter, L., Meier, H., Yadhukumar *et al.* (2004) ARB: a software environment for sequence data. Nucleic Acids Res 32: 1363-1371.

Martin, M. (2011) Cutadapt removes adapter sequences from high-throughput sequencing reads. EMBnet J 17: 10-12

Mezawa, T., Mochizuki, A., Miyakawa, K., and Sasamoto, H. (2018) Records of physico-chemical parameters by geochemical monitoring system in the Horonobe Underground Research Laboratory. URL: https://doi.org/10.11484/jaea-data-code-2018-001

Miyakawa, K., Ishii, E., Hirota, A., Komatsu, D.D., Ikeya, K., and Tsunogai, U. (2017) The role of low-temperature organic matter diagenesis in carbonate precipitation within a marine deposit. Appl Geochem 76: 218-231.

Miyakawa, K., Mezawa, T., Mochizuki, A., and Sasamoto, H. (2020) Data of groundwater chemistry obtained in the Horonobe Underground Research Laboratory Project (FY2017-FY2019). URL: https://doi.org/10.11484/jaea-data-code-2020-001

Nunoura, T., Takaki, Y., Kazama, H., Hirai, M., Ashi, J., Imachi, H., and Takai, K. (2012) Microbial diversity in deep-sea methane seep sediments presented by SSU rRNA gene tag sequencing. Microbes Environ 27: 382-390.

Pruesse, E., Quast, C., Knittel, K., Fuchs, B.M., Ludwig, W., Peplies, J., and Glöckner, F.O. (2007) SILVA: a comprehensive online resource for quality checked and aligned ribosomal RNA sequence data compatible with ARB. Nucleic Acids Res 35: 7188-7196

Pruesse, E., Peplies, J., and Glöckner, F.O. (2012) SINA: accurate high-throughput multiple sequence alignment of ribosomal RNA genes. Bioinformatics 28: 1823-1829

Quast, C., Pruesse, E., Yilmaz, P., Gerken, J., Schweer, T., Yarza, P. *et al.* (2013) The SILVA ribosomal RNA gene database project: improved data processing and web-based tools. Nucleic Acids Res 41: D590-D596.

Teramoto, M., Shimada, J., and Kunimaru, T. (2006) Evidences of groundwater regime in impermeable rocks by stable isotopes in porewaters of drilled cores. J Jpn Soc Eng Geol 47: 68-76.

Trifinopoulos, J., Nguyen, L-T., von Haeseler, A., and Minh, B.Q. (2016) W-IQ-TREE: a fast online phylogenetic tool for maximum likelihood analysis. Nucleic Acids Res 44: W232-W235

Williamson, A.J., and Coates, J.D. (2016) Enrichment and isolation of metal respiring hydrocarbon oxidizers. In Hydrocarbon and Lipid Microbiology Protocols. McGenity, T.J., Timmis, K.N., and Nogales, B. (eds). Berlin: Springer, pp. 143-164
